# Stromal score as a prognostic factor in primary gastric cancer and close association with tumor immune microenvironment

**DOI:** 10.1002/cam4.2801

**Published:** 2020-05-20

**Authors:** Min Mao, Qingliang Yu, Rongzhi Huang, Yunxin Lu, Zhen Wang, Liang Liao

**Affiliations:** ^1^ First Clinical Medical College Guangxi Medical University Nanning China; ^2^ Department of Orthopedic Surgery The Tenth Affiliated Hospital of Guangxi Medical University Qinzhou First People's Hospital Qinzhou China; ^3^ Department of Gastrointestinal Surgery The First Affiliated Hospital of Guangxi Medical University Nanning China; ^4^ Department of Traumatic Orthopedics and Hand Surgery The First Affiliated Hospital of Guangxi Medical University Nanning China

**Keywords:** estimate algorithm, primary gastric cancer, stromal score, tumor microenvironment

## Abstract

**Background:**

Gastric cancer remains one of the major causes for tumor‐related deaths worldwide. Our study aimed to provide an understanding of primary gastric cancer and prompt its clinical diagnosis and treatment.

**Methods:**

We integrated the expression profiles and overall survival information of primary gastric cancer in TCGA and GEO database and estimated the stromal score of each sample by the estimate R package. Stromal score and clinicopathologic characteristics associated with overall survival were analyzed by using Cox regression and the Kaplan‐Meier method. Gene set enrichment analysis (GSEA) and KEGG analysis were performed to explore the potential molecular mechanism in TCGA dataset. The relationship between immunotherapy‐associated markers or immune cell types and stromal score was explored by using Pearson correlation analysis.

**Results:**

A total of 796 samples were collected for the analysis. Patients with stromal score‐high showed poor overall survival (*P* < .01, HR: 1.407, 95% CI: 1.144‐1.731) and identified as an independent prognostic factor. KEGG analysis revealed that stromal score actively involved in diverse tumor‐associated pathways. GSEA analysis also revealed stromal score associated with diverse immune‐related biological processes. Furthermore, stromal score was related with immunotherapy‐associated markers and multiple immune cells.

**Conclusion:**

Our results showed that stromal score could serve as a potential prognostic biomarker in primary gastric cancer and play an important role in the recognition, surveillance, and prognosis of gastric cancer.

## Graphical abstract

Our study had shown that Stromal score could serve as a biological biomarker for primary gastric cancer and an independent prognostic factor. The prognosis mechanism of stromal score was associate with gene mutation, TMB, MATH, immunity and multiple signaling pathways. These findings may prompt the clinical diagnosis and treatment of primary gastric cancer.

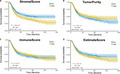

## 1 INTRODUCTION

Gastric cancer (GC) remains one of the primary reasons for tumor‐related deaths worldwide, despite its prevalence has dramatically fallen over the past decades.^1^, ^2^ Various risk factors contribute to the progression of gastric cancer, whereas Helicobacter pylori infection is the most definite risk factor.^3^ A majority of GC patients are diagnosed at an advanced and fatal stage. Currently, surgical resection is the major therapy for gastric cancer, with poor overall 5‐year survival rate (approximately 20%‐25%).^4^ Moreover, approximately half of the patients appear local or systemic tumor recurrence after adjuvant therapy.^4^ Therefore, novel therapeutic strategies and biomarkers are urgently acquired to improve the prognosis of gastric cancer.

Growing evidence revealed tumor microenvironment (TME), an aggregation of tumor cells and neighbouring tumor‐related nontumor cells, is crucial in tumor biology.^5^ A large number of researches supported tumor‐related stroma participated in the progression, metastasis of tumor, and the response to chemotherapy.^6^ Stromal cells are one of the pivotal components of TME, and the percentage of stromal cells in TME represents the stromal score.^7^ Tumor stroma, particularly the cell components are critical in the development of pancreatic ductal adenocarcinoma.^8^ Prostate stroma plays a significant role in the growth and differentiation of the normal prostate cells and has strong relationships with the occurrence of prostatic cancer and prostatic hypertrophy.^9^ These studies indicate that tumor immune microenvironment plays a crucial role in the development of numerous cancers. ESTIMATE algorithm could infer TME score by expression profiles data, such as stromal score, immunity score, and tumor purity.^10^ Additionally, it may be important in improving some defects including the visual effect of hematoxylin‐eosin staining, the method of computer‐aided estimate in the proportion of tumors and stromal. Currently, there are some reports on the use of ESTIMATE algorithm in colon cancer, breast cancer, and other tumors.^11^, ^12^ However, the role of ESTIMATE algorithm in GC remains to be elucidated.

In this study, the estimate algorithm was used to predict the stromal score of primary gastric cancer. We aimed to systematically explore stromal score and prognostic landscape and develop an individualized prognostic biomarker in primary gastric cancer. We further used bioinformatics analyses to explore underlying mechanisms of stromal score. Results from this study could offer foundation for subsequent personalized diagnosis and treatment of gastric cancer.

## 2 METHODS

### 2.1 Data processing

RNA‐Seq and clinical data of primary gastric cancer were acquired from TCGAbiolinks R package. Additionally, the expression profiles of GSE15459 and GSE62254 were downloaded by GEOquery R package. In this study, the samples with full data for cancer stage and follow‐up time were included, with simultaneous follow‐up time beyond 30 days. Next, the Ensembl IDs were converted into gene symbol through a matrix of gene symbols through the Ensembl database (http://asia.ensembl.org/index.html) in TCGA dataset. The Probe IDs were also transferred to gene symbol by hug133plus.db R package. The results were averaged when one patient had a matched multiple transcriptomics profile. The probe with maximum mean was reversed when more than one probe had a matched gene name.

### 2.2 Survival outcomes and multivariate COX analysis

The stromal score, immune score, tumor purity, and estimate score of each included sample was calculated by applying Estimate R package. Kaplan‐Meier curve analysis was further conducted to evaluate the relationship of these scores with overall survival. The cut‐off value was determined by its median value. To identify, the index was an independent factor, univariate and multivariate Cox regression analysis was performed in combination with clinical indicators.

### 2.3 Stromal score and genomic analysis

To explore the potential mechanism of the prognostic value of stromal score, RNA‐Seq data and gene mutation maf data were downloaded through the TCGAbiolinks R package. The relationship between the mutant allele tumor heterogeneity (MATH) or tumor mutation burden (TMB) and stromal score was analyzed through the Wilcoxon test. We employed Chi‐Square test to identify differential mutated genes in both groups and conducted KEGG analysis for the prediction of conceivable pathways of differential mutant genes. To identify signaling biological processes that are differentially activated between the high and low stromal score group in the TCGA dataset, we identified an ordered list of genes through edgeR R package and conducted Gene set enrichment analysis (GSEA) on the gene with adjusted *P* < .05.

### 2.4 Stromal score and tumor microenvironment

CIBERSORT could be used to estimate the fraction of 22 immune cell types of Tumor microenvironment through using a machine‐learning approach called support vector regression.To further evaluate the prognostic value of the stromal score, the relative components of 22 leukocytes were calculated for each sample of three datasets in the R platform. The correlation between each component and stromal score was calculated by the Hmisc R package. *P* < .05 and/R/ >0.12 indicated that correlation existed between the stromal score and its component. These components were divided into two groups high and low group based on their median value and performance with KM analysis.

### 2.5 Statistical analysis

All statistical analyses were performed in R 3.5.2 (http://www.r-project.org/) and its corresponding R package. The KM analysis and Cox regression analyses were completed by using the survival R package. The relationship between the stage clinical pathologic features and the stromal score was analyzed with the Kruskal‐Wallis test. Maf mutation data were analyzed and summarized by utilizing the maftools R package. The relationship between immunotherapy‐associated markers (PD‐1L, CTLA‐4, LAG‐3, TIM‐3, PD‐1) and the stromal score was explored by using Pearson correlation analysis in TCGA dataset.The c5.bp.v6.2.entrez.gmt file came from the Molecular Signatures Database (MSigDB, http://software.broadinstitute.org/gsea/index.jsp) was downloaded for the GSEA analysis. GSEA and KEGG analysis were completed by using the clusterprofiler R package.

## 3 RESULTS

### 3.1 Data processing

After data screening, a total of 796 primary gastric cancer samples were included in the analysis. The detailed clinical characteristics of all enrolled patients were presented in Table 1.

**Table 1:** The clinical characteristics of all enrolled patients

Characteristics	Group	Overall
Total		796
Age		63.69 (11.43)
Gender	F	281 (35.3)
	M	515 (64.7)
Stage	I	103 (12.9)
	II	229 (28.8)
	III	298 (37.4)
	IV	166 (20.9)
StromalScore		180.97 (871.28)
ImmuneScore		972.16 (823.11)
EstimateScore		1153.13 (1552.57)
TumorPurity		0.70 (0.16)
Overall Time(Month)		37.12 (35.32)
Overall Status	Alive	437 (54.9)
	Dead	359 (45.1)

Note

Table 1 shows the mean value and standard deviation of age, stromal score, immune score, and EstimateScore, TumorPurity, Overall Time, as well as the number and proportion of gender stage and survival status.

### 3.2 Stromal score and stage clinical characteristics

Further analysis found that the stromal score was apparently correlated with the TNM Stage. Stromal score rose progressively with TNM staging, whereas such a trend did not arise in Stage III and IV.

### 3.3 Survival outcomes and multivariate COX analysis

**Figure 1:**
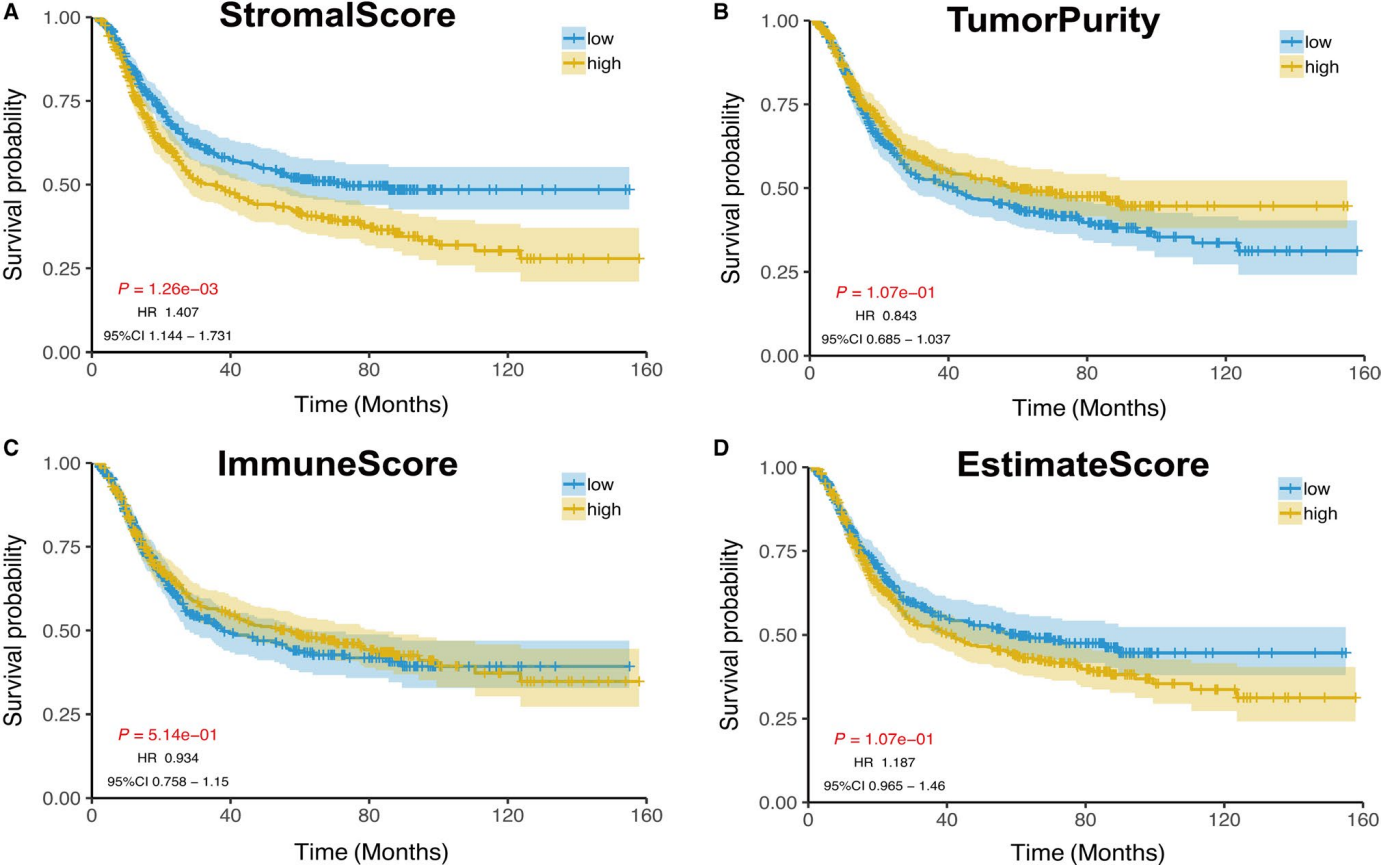
Impact of stromal score on overall survival in primary gastric cancer based on KM analysis. The cut‐off value was determined by its median value. A, Stromal score. B, Tumor purity. C, Immune score. D, Estimate score

As shown in Figure 1, stromal score had significant statistical difference in distinguishing the high‐ and low‐score groups of primary gastric cancer. Patients in high‐score group showed poor overall survival (*P* < .01, HR: 1.407, 95% CI: 1.144‐1.731). Inversely, in both groups, no statistically significant difference among tumor purity, immune score, and estimate score was noted. The univariate analysis revealed that stromal score highly correlated significantly with a poor OS (HR: 1.409; 95% CI: 1.143‐1.736; *P* = 1.33e‐3). Stage was also associated with poor survival (Table 2).

**Table 2:** The results of univariate and multivariable Cox regression analyses

Characteristics	Univariate analysis		Multivariable analysis	
	HR(95% CI)	*P*	HR(95% CI)	*P*
Age				
≤60	Reference		Reference	
>60	1.323 (1.059‐1.653)	1.36e‐02	1.598 (1.276‐2.001)	4.51e‐05
Gender				
M	Reference			
F	0.893 (0.716‐1.114)	3.16e‐01		
Stage				
I	Reference		Reference	
II	1.781 (1.071‐2.963)	1.55e‐01	1.462 (1.048‐2.899)	3.24e‐02
III	3.595 (2.227‐5.803)	6.88e‐07	3.473 (2.086‐5.782)	3.88e‐07
IV	7.981 (4.916‐12.957)	5.09e‐16	8.575 (8.297‐13.495)	1.53e‐17
Stromal score				
Low	Reference		Reference	
High	1.409 (1.143‐1.736)	1.33e‐03	1.308 (1.059‐1.616)	1.29e‐02
Immune score				
Low	Reference			
High	0.933 (0.757‐1.149)	5.15e‐01		
ESTIMATE score				
Low	Reference			
High	1.187 (0.963‐1.463)	1.08e‐01		
Tumor purity				
Low	Reference			
High	0.842 (0.684‐1.038)	1.08e‐01		

**Figure 2:**
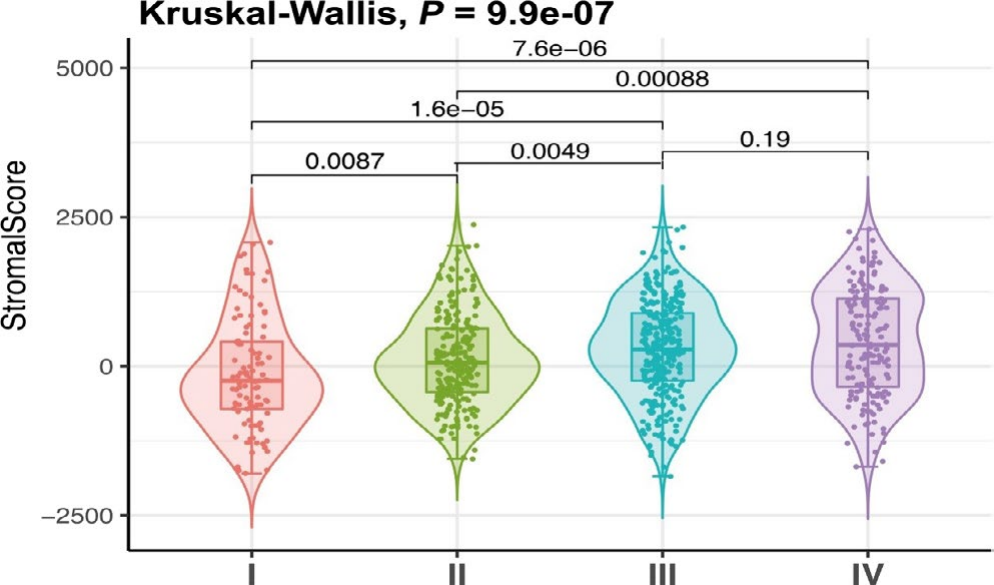
Association with stromal score and clinicopathologic stage characteristics based on Kruskal‐Wallis test

**Figure 3:**
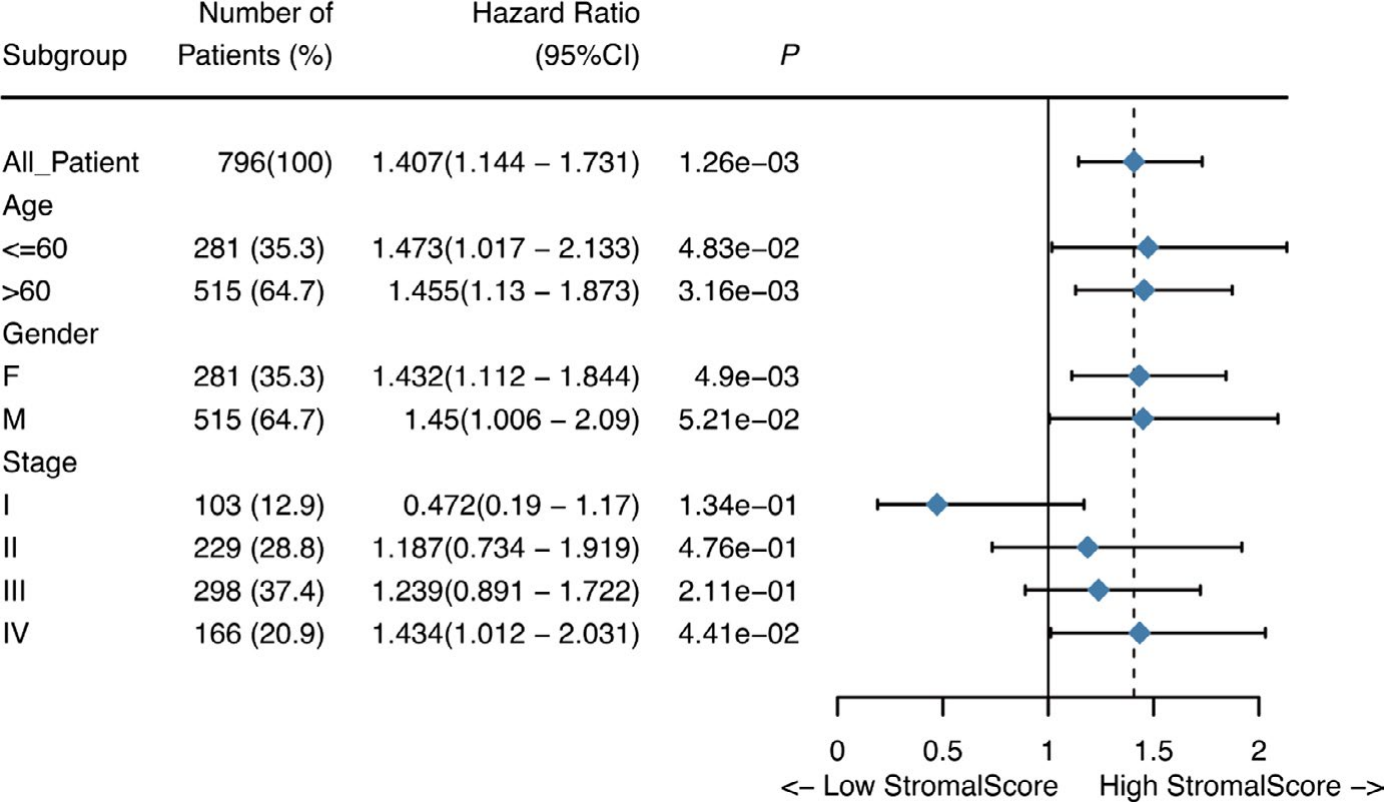
The forest map showed the results of subgroup analysis

Subgroup analyses revealed that high stromal score impaired survival in stage IV (*P* = 4.41e‐2) and age (*P*(>60) = 3.16e‐3 and *P*(≤60) = 4.83e‐2) and gender (*P*(female) = 4.9e‐3, *P*(man) = 5.21e‐2). At multivariate analysis, stromal score remained independently associated with overall survival, with a HR of 8.575 (CI: 1.059‐1.616, *P* = 1.29e‐2) along with other features (Figures 2 and 3).

### 3.4 Stromal score and mutant genomic analysis

**Figure 4:**
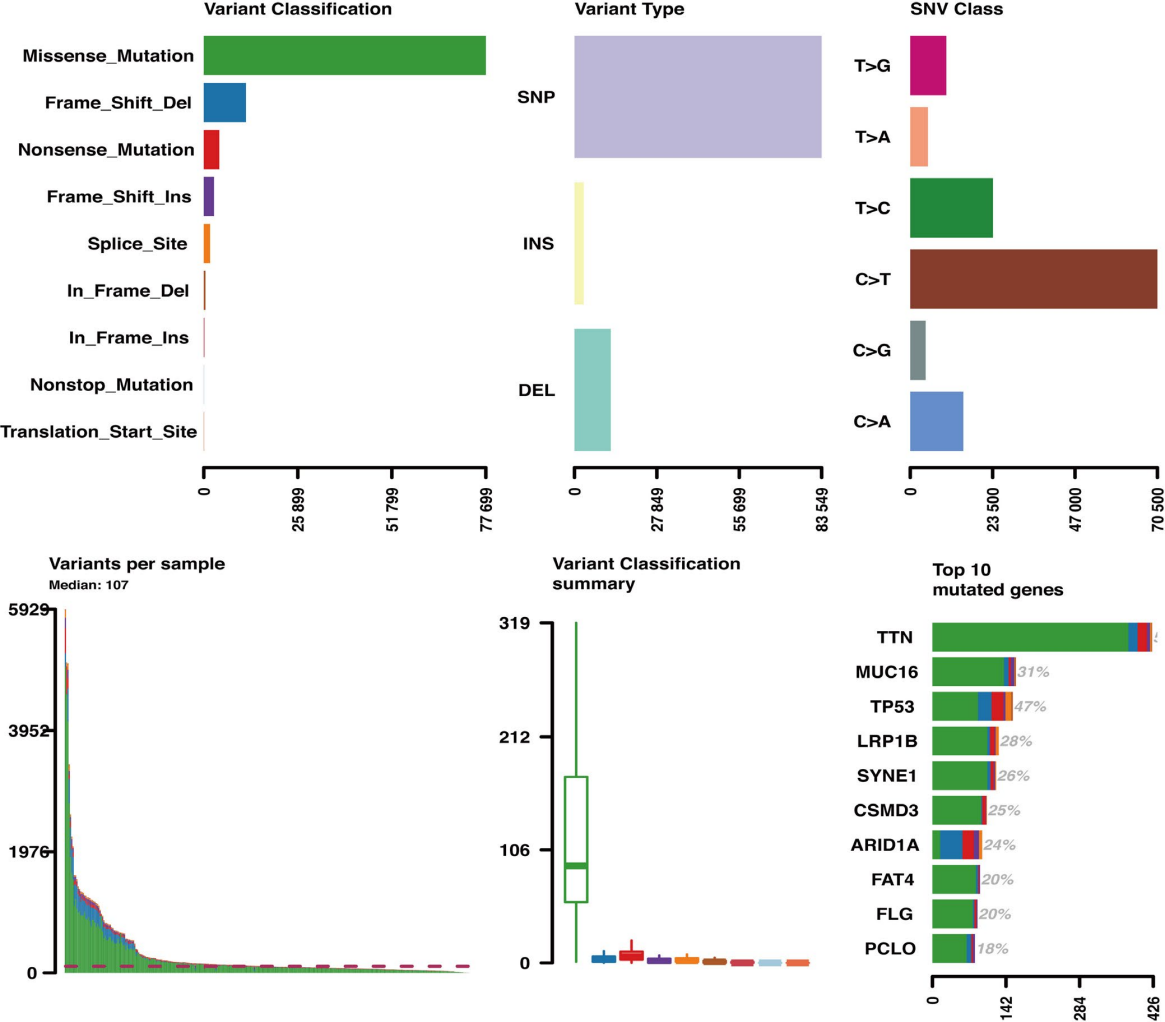
General overview of mutation in primary gastric cancer

**Figure 5:**
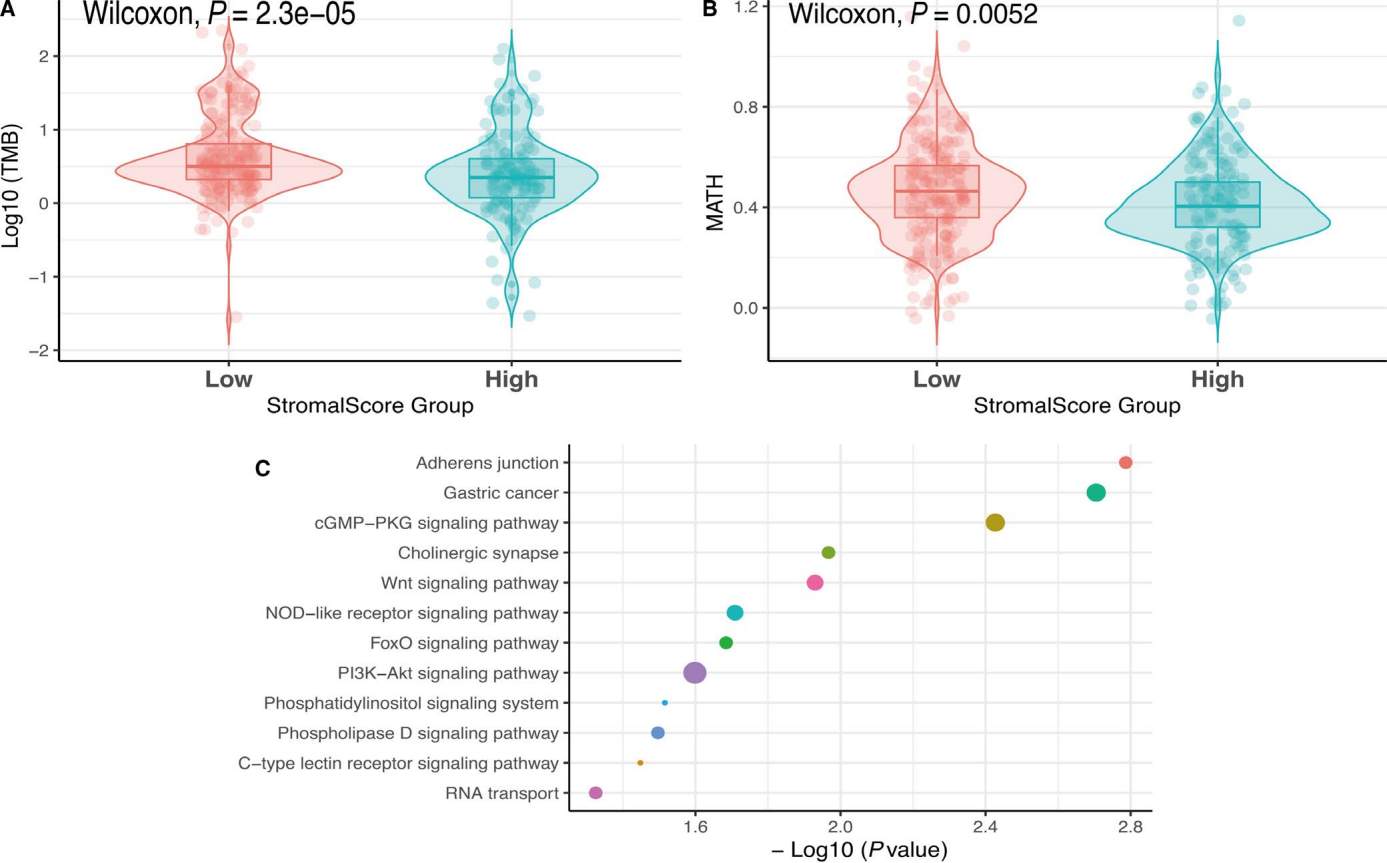
A, Association with stromal score and TMB (tumor mutation burden). B, Association with stromal score and MATH (mutant allele tumor heterogeneity). C, Parts of results of KEGG analysis

C > T mutation was the most frequent SNV mutation type, and TTN gene mutation rates are the highest in primary gastric cancer (Figure 4). Tumor mutation burden (TMB) and mutant allele tumor heterogeneity (MATH) are different between high‐ and low‐score groups based on the Wilcoxon test. Assessed by the Chi‐squared test, 215 mutant genes were identified to have significant differences in low and high stromal score groups according to the criterion of *P* < .05 (S1). KEGG pathway analysis revealed that mutant genes enriched in diverse tumor signal pathways, including the PI3K‐Akt signaling pathway, the cGMP‐PKG signaling pathway, the Wnt signaling pathway, the NOD‐like receptor signaling pathway, and other pathways (Figure 5C, Figure S2).

### 3.5 Stromal score and RNA‐Seq genomic analysis

**Figure 6:**
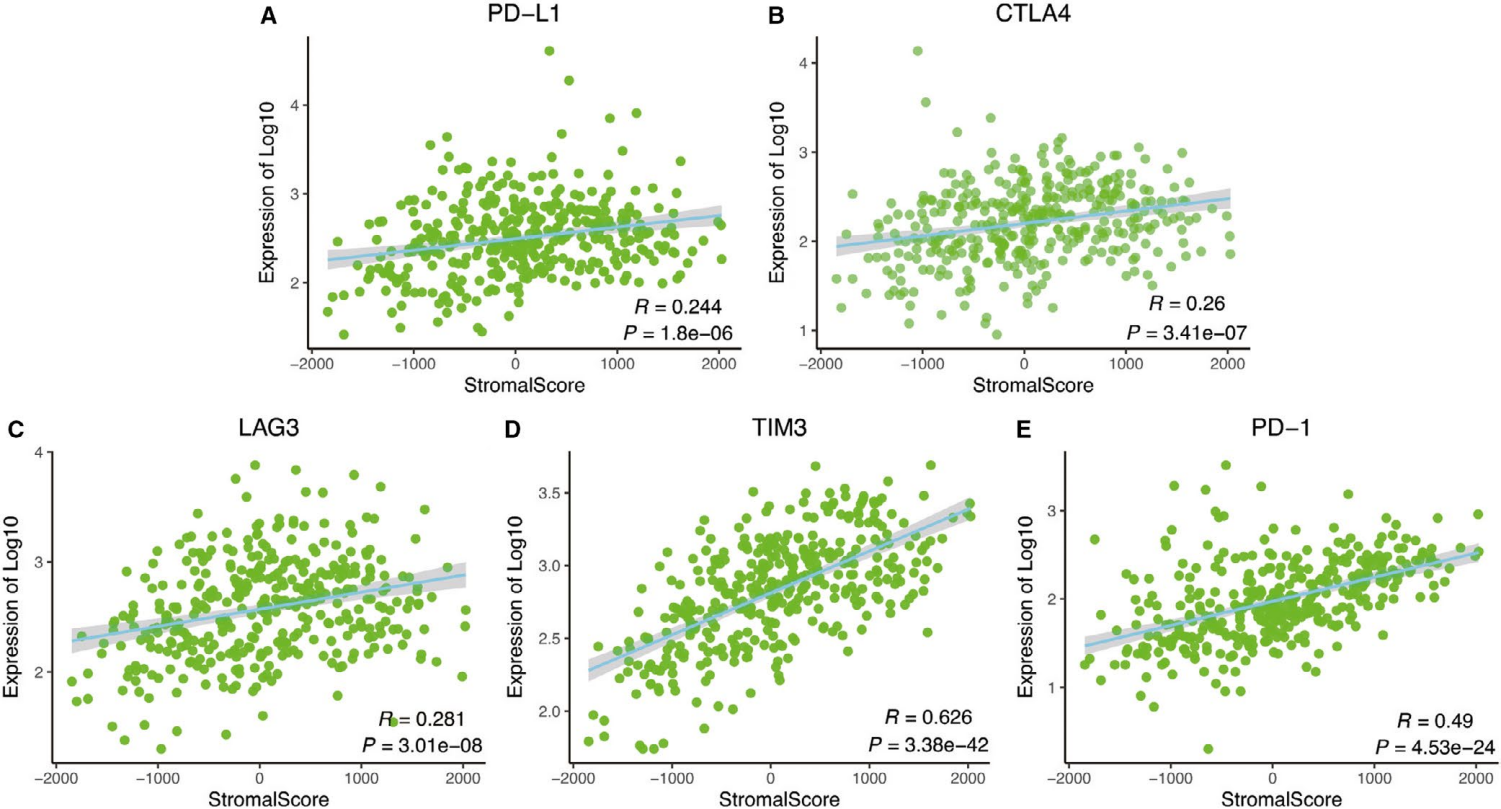
The relationship between stromal score and immunotherapy‐associated biomarkers based on correlated analysis in RNA‐Seq data of TCGA

**Figure 7:**
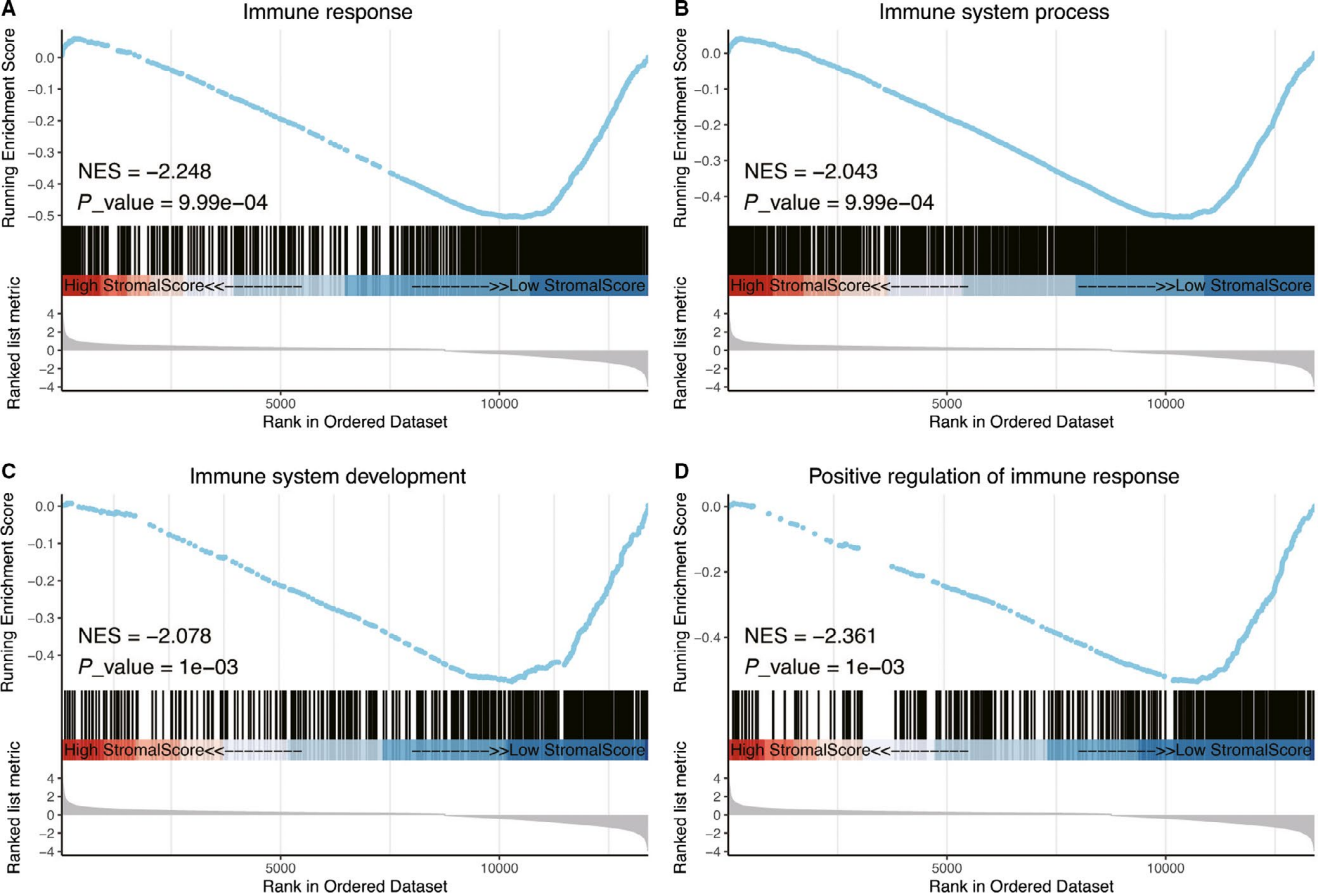
Parts of the results of GSEA in the TGCA dataset

The expression level of immunotherapy‐associated markers (PD‐L1, CTLA4, LAG3, TIM3, PD‐1) was significantly associated with the stromal score in RNA‐Seq data of TCGA (Figures 6A‐E). Various immune‐associated biological processes were enriched, including immune response, immune system process, immune system development, positive regulation of immune response, and others (Figure 7A‐D, Table S3).

### 3.6 Stromal score and tumor microenvironment

**Figure 8:**
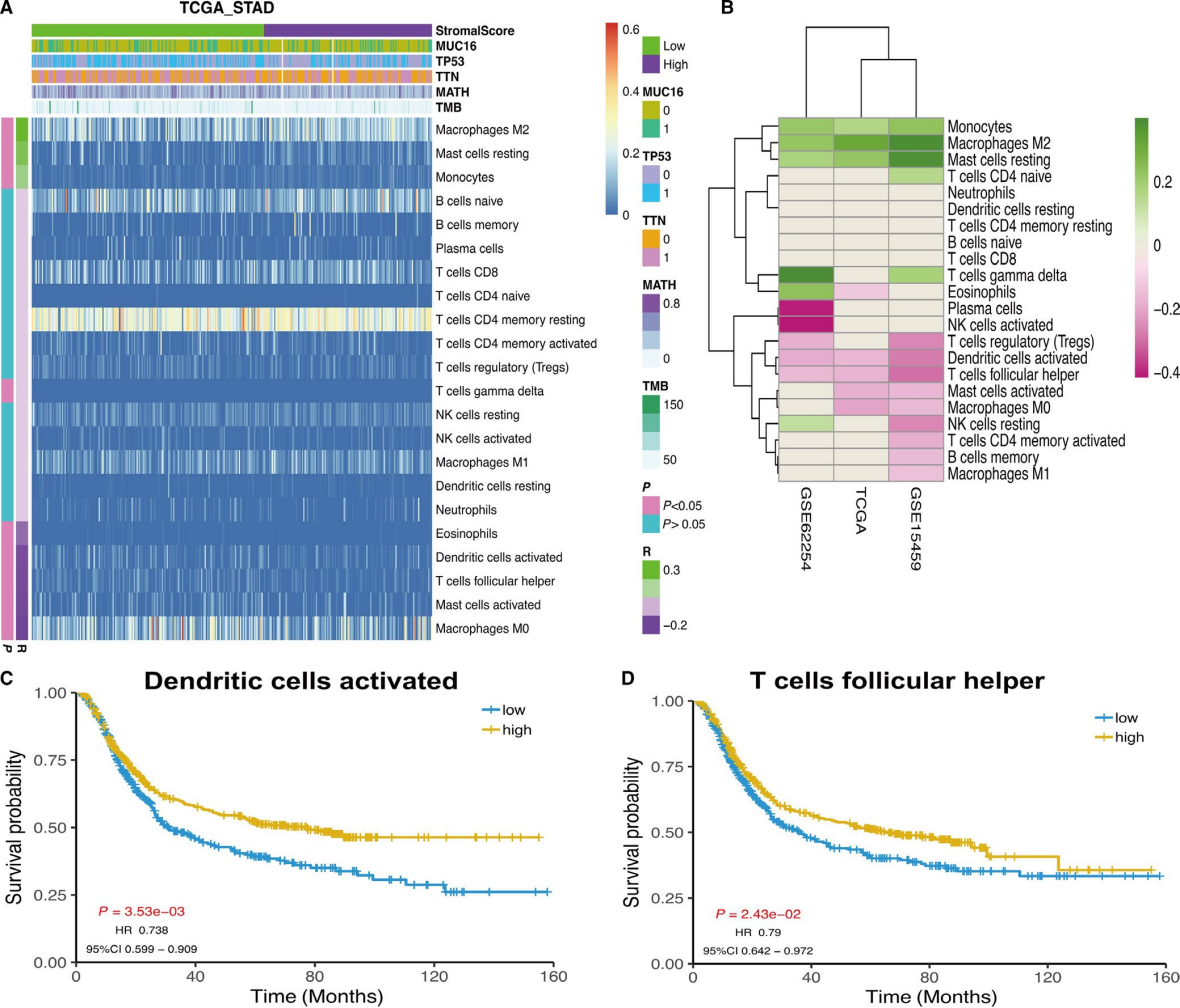
A, The heap map of the relative proportions of immune cells. The left color bar indicated the correlation coefficients and *P* value. B, The correlation coefficients of immune cells and stroma score in TCGA, GSE15459, and GSE62254 datasets. C, The KM analysis result of relative proportion of activated dendritic cells. D, The KM analysis of relative proportion results of T cells follicular helper

The correlations between differential relative proportions of immune cells and stromal score were plotted in Figure 8A, Figure 1 and S2. The correlation coefficients of the relative proportion of immune cell and stromal score were drawn in the heat map. Activated dendritic cells and T cells follicular helper cells were a negative correlation to the coefficients in three datasets (Figure 8B). In Kaplan‐Meier survival analysis, Dendritic cells activated and T cells follicular helper were markedly related to overall survival of primary gastric cancer. The low‐level group of two kinds of cells had a worse survival compared with the high‐level group (*P* < .05, Figure 8C,D).

## 4 DISCUSSION

In this study, we identified the stroma score could serve as a potential prognosis biomarker and explored the molecular mechanism in gastric cancer through comprehensive analysis. Stromal score impacted the overall survival in primary gastric cancer. A high stromal score has a poor OS in this study. Moreover, stromal score was associated with the pathological tumor stage. High pathological stage presented a high‐level stromal score, which predicated a high level stromal score and a poor prognosis. High TNM stage presented high‐level stromal score, predicating poorer prognosis.

Previously, several studies had reported tumor stromal strongly implicated in facilitating the growth, differentiation, progression, and metastasis of malignancies by nourishing tumor parenchyma.^13^, ^14^ Stromal cell ratio within the tumor microenvironment (TME) has been certified as an independent prognostic significance in the assessment of cancer therapy, whereas a high stromal cell ratio acted as a predictive factor for adverse outcomes in multiple malignancies.^15^ These findings confirmed stromal score had a tight correlation with clinical manifestations and prognosis of malignancies, and suggested that stromal score could act as a provital prognostic factor for tumors. In this research, stromal score had predictive value in primary gastric cancer to a certain extent.

To explore the mechanism of between stromal score and overall survival in primary gastric cancer, we conducted multiple Computational analyses. We found the difference in MATH and TMB between the high and the low stroma score group. MATH and TMB were served as tumor biomarkers in the assessment of prognosis of tumor in the past. Tumor mutation burden (TMB), a novel emerging biomarker in immunotherapy, presented the total index of mutations in tumor samples.^16^ It was proved to be related to the responses to PD‐1/PD‐L1 blockade in multiple tumors, including urothelial carcinoma, non‐small cell lung cancer (NSCLC), colorectal cancer, and melanoma.^17^ Mutant allele tumor heterogeneity (MATH) is a new, nonbiased, quantitative approach to evaluate genetic heterogeneity according to tumor next generation exome sequencing.^18^ MATH score had a strong correlation with tumor stage by calculating and comparing the MATH scores of colon cancer samples.^19^ Based on previous researches and this analysis, we further determined the effect of stromal score on overall survival of gastric cancer.

A large number of differential gene mutations and various tumor‐related pathways acted as important roles in the molecular mechanism of stromal score. The PI3K‐Akt pathway and cGMP‐PKG signaling pathway were the key pathways in KEGG analysis. These results were consistent with previous studies. Activation of PI3K‐Akt pathway could take part in protecting gastric mucosal epithelium against damage.^20^ In cancer patients, multiple components of the PI3K‐Akt signaling pathway were amplified, mutated, and translocated more frequently compared with other pathways, resulting in pathway activation.^21^ cGMP‐PKG signaling pathway involved in the treatment and prevention of numerous cancers, including cervical cancer, colon cancer, and breast cancer.^22^, ^23^, ^24^ In the GSEA analysis, we also found many immune‐related biological processes taking part in the molecular mechanism. These immune‐related biological processes included immune response, immune system process, immune system development, and positive regulation of immune response. Our results revealed that stromal score took part in immune regulation of primary gastric cancers.

In the correlation analysis between stromal score with immunotherapy‐associated markers, we also found that stromal score was associated with the expression of PD‐L1, CTLA4, LAG3, TIM3, PD‐1. These results also indicated that stromal score played a role in immune regulation of gastric cancer. CTLA‐4, PD‐1 and some other immune checkpoint molecules participated in the inhibiting activation of T cell by different pathways.^25^ The studies concerning CTLA‐4 mainly focused on breast cancer and inflammation, whereas the study on gastric cancer focused on deficiency. The expressions of PD‐L1 molecule in gastrointestinal malignancies and gastric cancer were approximately 20%‐55% and 40%, respectively.^26^ And PD‐1 molecule expression on CD4^+^ and CD8^+^ T cells in the gastric cancer was dramatically higher as compared with normal gastric mucosa.^27^ Connection between PD‐L1/PD‐1 expression and diverse clinicopathological characteristics may serve as an alternative marker of PD‐L1+ gastric cancer, improving the possibilities of immune checkpoint treatment, simultaneously.^28^ Lymphocyte‐activation gene 3 (Lag3, CD223) expresses itself on diverse immune cells 29 and has multiple biologic functions, such as functions as a negative regulator for proliferation, activation of T cells, and homeostasis.^30^ Recently, Junlong Wu et al found that low TIM3 expression revealed a poor prognosis in metastatic prostate cancer.^31^ Similarly, little work has been performed on the role of Lag3 and TIM3 in GC. This study gives us some new insight into immunotherapy and monitoring in gastric cancers.

Relative proportion of Dendritic cells and T cells follicular helper cells were negatively correlated with stromal score in all datasets. It was not difficult to find the proportion that acted as a protective factor, suggesting that the stromal score was a risk factor in primary gastric cancer. Currently, a large number of researches have proved various immune cells involved in the progression of GC. Tumor‐associated macrophages were considered to support the growth and metastasis of GC and were positively related to its invasion depth and clinical stage.^32^ Besides, CD3^+^ and CD8^+^ T lymphocytes with high infiltration have a connection with favorable outcomes in GC, suggesting the significant role of host immunity mediated by T cells in restraining tumor progression.^2^, ^33^ Consistent with the function of CD3^+^ and CD8^+^ T lymphocytes, activated dendritic cells and T cells follicular helper were anti‐tumor components associated with stromal score in our analysis. Unfortunately, the two kinds of cells have not been reported in gastric cancer. These preliminary analysis, results, and observation could provide a perspective to explore problems.

This research comprehensively explored the association in stromal score, clinical characteristics, and prognosis of gastric cancer in R platform. Meanwhile, we investigated the latent mechanism of stromal score by applying multiple analytical methods and genomic analyses. However, some limitations should be considered in this study. First, this study was a retrospective study. And prospective study was needed. Second, the stromal score was calculated only based on transcriptome data. Stromal score inferred by combining with multiple genomic data, such as mutant and methylation, could improve credibility. Third, the reliability of our molecular mechanism analysis results was challenged because of the lack of in vitro or in vivo experiments.

## 5 CONCLUSION

We anticipate that the stromal score could serve as a potential prognostic biomarker that systematically analyzed the role of stromal score in the monitoring of prognosis in primary gastric cancer. The findings provide novel insights into the monitoring and treatment of gastric cancer.

## Data Availability

We downloaded GSE15459 and GSE62254 gene expression profiles from GEO (https://www.ncbi.nlm.nih.gov/geo). The c5.bp.v6.2.entrez.gmt file came from Molecular Signatures Database (MSigDB, http://software.broadinstitute.org/gsea/index.jsp) was downloaded for the GSEA analysis. All statistical analyses were performed in R 3.5.2 (http://www.r-project.org/) and its corresponding R package.
